# In Vitro Gastrointestinal Digestion of Various Sweet Potato Leaves: Polyphenol Profiles, Bioaccessibility and Bioavailability Elucidation

**DOI:** 10.3390/antiox13050520

**Published:** 2024-04-26

**Authors:** Junren Wen, Yong Sui, Jianbin Shi, Sha Cai, Tian Xiong, Fang Cai, Lei Zhou, Shuyi Li, Xin Mei

**Affiliations:** 1 Key Laboratory of Agro-Products Cold Chain Logistics, Ministry of Agriculture and Rural Affairs, Institute of Agro-Products Processing and Nuclear-Agricultural Technology, Agro-Product Processing Research Sub-Center of Hubei Innovation Center of Agriculture Science and Technology, Hubei Academy of Agricultural Science, Wuhan 430064, China; jasminewen@webmail.hzau.edu.cn (J.W.); shijianbin@hbaas.com (J.S.); caisha@hbaas.com (S.C.); xiongtian@hbaas.com (T.X.); hmshi90@hbaas.com (F.C.); zhouleidjj@163.com (L.Z.); 2 College of Food Science and Technology, Huazhong Agricultural University, Wuhan 430070, China; 3 National R & D Center for Se-Rich Agricultural Products Processing, School of Modern Industry for Selenium Science and Engineering, Wuhan Polytechnic University, Wuhan 430023, China; shuyi.li@whpu.edu; 4 Hubei Engineering Research Center for Deep Processing of Green Se-Rich Agricultural Product, School of Modern Industry for Selenium Science and Engineering, Wuhan Polytechnic University, Wuhan 430023, China

**Keywords:** sweet potato leaves, caffeoylquinic acid, phenolic profile, bioaccessibility, bioavailability

## Abstract

The chemical composition discrepancies of five sweet potato leaves (SPLs) and their phenolic profile variations during in vitro digestion were investigated. The results indicated that Ecaishu No. 10 (EC10) provided better retention capacity for phenolic compounds after drying. Furthermore, polyphenols were progressively released from the matrix as the digestion process proceeded. The highest bioaccessibility of polyphenols was found in EC10 intestinal chyme at 48.47%. For its phenolic profile, 3-, 4-, and 5-monosubstituted caffeoyl quinic acids were 9.75%, 57.39%, and 79.37%, respectively, while 3,4-, 3,5-, and 4,5-disubstituted caffeoyl quinic acids were 6.55, 0.27 and 13.18%, respectively. In contrast, the 3,4-, 3,5-, 4,5-disubstituted caffeoylquinic acid in the intestinal fluid after dialysis bag treatment was 62.12%, 79.12%, and 62.98%, respectively, which resulted in relatively enhanced bioactivities (DPPH, 10.51 μmol Trolox/g; FRAP, 8.89 μmol Trolox/g; ORAC, 7.32 μmol Trolox/g; IC50 for α-amylase, 19.36 mg/g; IC50 for α-glucosidase, 25.21 mg/g). In summary, desirable phenolic acid release characteristics and bioactivity of EC10 were observed in this study, indicating that it has potential as a functional food ingredient, which is conducive to the exploitation of the sweet potato processing industry from a long-term perspective.

## 1. Introduction

Sweet potato (*Ipomoea batatas* L.) leaves (SPLs), as an edible and fast-growing plant belonging to the Convolvulaceae family [1], have been gaining importance in recent years as there is increasing consumer demand for healthy and sustainable food [2]. Many previous studies have reported that SPLs are associated with promising bioactivities, including antioxidant, antidiabetic, antitumor and anti-inflammatory capacity, and protecting hepatic and/or cardiac lesions [3]. SPLs used to be discarded as a by-product in the sweet potato processing industry and only approximately 10% of the annual production is now being consumed as a new type of healthy vegetable in several countries [4]. Obviously, the lack of deep processing products of SPLs still exists [5], and only Toy et al. have reported in recent years that they successfully extracted and modified the pectin from SPLs with enhanced biological activities [6]. Those products could be utilized as a functional food ingredient, which partly broadened the range of applications of SPLs, whereas the application of phenolic acids from SPLs (mainly derivatives of caffeic and quininic acid) in foods was still unreported, and few references were focused on describing the changes in polyphenol profiles during digestion and the beneficial effects of SPLs as a food ingredient.

Research suggested that the biological activity of SPLs was attributed to the promising polyphenol content—in particular, caffeoylquinic acid exhibited favorable antioxidant activity in vitro [1,7]. As a prominent indicator for the evaluated ability of polyphenols to enter the gastrointestinal (GI) system after being released from the food substrate, further through the circulatory system to reach the target organ [8], bioaccessibility was essential for assessing the biological relevance of phytochemicals to human health. Previous studies have shown that consuming polyphenols through oral intake from leafy vegetables may not achieve the desired results [9]. This is because the physicochemical properties and stability of polyphenols and their bioactivity may vary during the oral-GI digestion stage, which is contingent upon the effects caused by pH, temperature, metal ions intensity and the presence of digestive enzyme system [10]. Examples include a study on the bioavailability of mulberry leaves, which showed that the absorption of polyphenols during digestion in the GI tract was generally low [11], while unencapsulated anthocyanins from red cabbage were similarly hypersensitive in the neutral pH of intestine environments, resulting in phenolic acid degradation and low bioaccessibility recorded by Izzo et al. [12]. Since the phenolic profiles of various leafy vegetables were distinctive, this results in diverse phenolic acid release characteristics during GI digestion. However, as far as we were concerned, there were limited reports on phenolic profile changes, releasing characteristics and bioactivity in SPLs by the simulated digestive system in vitro.

In summary, our study initially clarified the chemical composition of five varieties of SPL samples that have been widely cultivated in China. The freeze-dried SPLs were further investigated in simulating an oral-GI digestion model coupled with UPLC-HRMS in terms of their (1) polyphenol profiles, bioaccessibility and bioavailability changes; (2) antioxidant and α-amylase/α-glucosidase inhibitory activities of the phenolic during the digestive process in vitro. By understanding the digestive release behavior of sweet potato leaf polyphenols (SPLPs) and their potential health benefits to humans, functional foods based on SPLPs with superior bioactive potential could be exploited, which provides other solutions for the intensive processing and utilization of SPLs.

## 2. Materials and Methods

### 2.1. Chemicals and Materials

Fushu No. 18 (F18, National sweet potato identification code. 2011015), Ningcaishu No. 1 (NC1, National sweet potato identification code. 2013014), Xucaishu No. 1 (XC1 National sweet potato identification code. 2013015), Fushu No. 23 (F23, National sweet potato identification code. 2016030) and EC10 (National sweet potato identification code. 2013014) were obtained from Food Crops Institute, Hubei Academy of Agricultural Sciences (114°19′3.87″ E, 30°28′56.92″ N), and identified by Fujian Academy of Agricultural Sciences. SPLs were washed, ironed, bleached, freeze-dried and milled for subsequent experiments. Since the high moisture content in fresh samples of SPLs could be unfavorable for storage and milling, the control check (CK) was the corresponding SPLs frozen in liquid nitrogen to be milled into homogeneous powder before the simulated digestion to prevent bias in the results.

2,6-Dichloroindophenol, *p*-Nitrophenyl-α-d-glucopyranoside (pNPG), ascorbic acid, pancreatin and bile salt were purchased from Yuanye Bio-Technology Co., Ltd. (Shanghai, China). Chlorogenic acid (CG), 1,1-Diphenyl-2-picrylhydrazyl (DPPH), 2,4,6-Tri(2-pyridyl)-s-triazine (TPTZ), 2,2′-Azobis(2-methylpropionamidine) dihydrochloride (AAPH), fluorescein sodium salt, Trolox, α-amylase (EC3.2.1.1) and pepsin (EC3.4.23.1) were purchased from Sigma-Aldrich (Shanghai) Trading Co., Ltd. (Shanghai, China). Chromatographic-grade methanoic acid, methanol and acetonitrile were acquired by Fisher Scientific (Waltham, MA, USA). Other analytical-grade chemical reagents and materials were supplied by Sinoreagent (Shanghai, China).

### 2.2. Chemical Composition Analysis

The content of moisture (Method AOAC 925.09), ashes (Method AOAC 923.03), crude protein (Method AOAC 954.01) and crude fat (Method AOAC 979.09) of the SPLs were exhaustively measured. A nitrogen coefficient of 6.25 was used to calculate protein contents.

#### 2.2.1. Ascorbic Acid Content (AAC) and Total Carbohydrate Content

AAC in SPLs was determined by 2,6-dichloroindophenol titration [13], and total carbohydrate content was measured by using the phenol-sulphonic acid method [14].

#### 2.2.2. Total Phenolic Content (TPC) and Total Flavonoid Content (TFC)

The extract was prepared by mixing 0.5 g in 20 mL of 70% ethanol (*v*/*v*). The mixture was ultrasonic extraction (KQ-500VDV, Kunshan Ultrasonic Instruments, Kunshan, China) for 30 min and the extraction procedure was repeated once, then decompression distillation was used to obtain crude extracts.

The TPC was quantified by the Folin–Ciocalteu method [15], and the result was expressed in mg of chlorogenic acid equivalents per g (mg CGE/g); The TFC was examined using the aluminum chloride colorimetric method, and the result was expressed in mg of rutin equivalents per g (mg RE/g). The change in TPC after drying is assessed in this analysis by the “retention rate”, which is calculated as follows:

$$\mathrm{Retention}\;\mathrm{rate}\left(\%\right)=\frac{{BC}_{before}-{BC}_{after}}{{BC}_{before}}\times 100\%$$

where BC_before_ was the TPC in SPLs before drying, while BC_after_ was the corresponding content in dried samples.

### 2.3. Polyphenol Profile Analysis

The crude extracts were further liquid–liquid extracted by ethyl acetate. Briefly, three volumes of ethyl acetate were thoroughly mixed with the digestive solution and partitioned, the process was repeated three times. All upper phases were collected and then dried through evaporation, further dissolved by 4.5 mL methanol. All the sample solutions were kept under −20 °C.

The polyphenol profiles of SPLs were demonstrated by a Q Exactive Orbitrap mass spectrometer coupled to UltiMate 3000 UHPLC system (Thermo Scientific, San Jose, CA, USA), and the method was optimized base on Sun et al. [16]. Shim-pack C18 column (150 × 4.6 mm, 5 µm, Shimadzu Co., Kyoto, Japan) were used to separate SPLPs in liquid chromatography. The column temperature was set to 25 °C, the flow rate was 0.8 mL/min and 10 μL samples were injected. The gradient elution procedure was used as follows: Eluent A (0.1% formic acid, *v*/*v*), Eluent B (100% acetonitrile), 0.01–12 min (95–80% A), 12–25 min (80–65% A), 25–35 min (65–95% A). The ionization method used high-energy spark-induced breakdown ionization (HESI-II). The temperature of the ion source and capillary were 300 °C and 320 °C, respectively, while the sheath and auxiliary gas were 40 and 10 arbitrary units, respectively. MS data acquisition was performed in the full-scan/dd-MS^2^ mode; range *m*/*z* 100–1500. All the identified SPLPs compounds were quantified by a chlorogenic acid standard curve and then expressed in mg of chlorogenic acid equivalents per g (mg CGE/g).

### 2.4. Simulated Digestion

SPLs under were oral-GI digestion in vitro according to the INFOGEST protocol with minor modifications [17]. Simulated salivary fluid (SSF), simulated gastric fluid (SGF) and simulated intestinal fluid (SIF) were ready-made according to Minekus et al. [18].

In the oral digestion stage, SSF (5.6 mL) was mixed with 0.5 g SPLs, then 0.5 mL α-amylase (1500 U/mL), 0.3 M CaCl_2_ (20 μL) and 1.56 mL of distilled water were added. The mixtures were incubated at 37 °C on an orbital shaker with oscillation shielded from light (150 rpm) for 3 min. During the gastric digestion stage, the pH of oral chyme was adjusted to 3.0 by HCl (0.1 M) to blunt the enzyme activity, then mixed with SGF (6 mL), CaCl_2_ (10 μL), 1.28 mL pepsin (25,000 U/mL), then SGF was added to bring the total volume to 16 mL. The reaction system was incubated for 2 h in the same way. For the last stage, the gastric juice was first mixed with 2 mL of porcine bile salt (65 mg/mL), and the pH was increased to 7 with NaOH solution (2 M). SIF (8.8 mL), CaCl_2_ (32 μL) and 4 mL pancreatin solution (800 U/mL) were mixed and SIF was added to bring the total volume to 32 mL. The reaction system was subsequently transferred to a dialysis bag (MWCO: 3500 Da, mymbio, Beijing, China), and incubated in 500 mL PBS (pH = 7) at 37 °C for 2 h with constant stirring (300 rpm) under darkness [19]. The retained digests were freeze-dried and stored at −20 °C for further analysis. Samples of each digestion stage were centrifuged at 8000× *g* at 4 °C for 15 min, the supernatant was purified by liquid–liquid extraction with ethyl acetate, and the extraction was distilled to dryness under reduced pressure, then the phenolic compounds were re-dissolve by methanol and stored at −20 °C for further tests. The bioavailability and bioaccessibility of SPLPs were calculated by the following equation:

$$\mathrm{Bioaccessibility}\;\left(\%\right)=\frac{{\mathrm{SPLPs}}_{D}}{{\mathrm{SPLPs}}_{O}}\times 100\%$$

where SPLPs_D_ was the content of the polyphenol compounds in the supernatant after each digestion stage, while SPLPs_O_ was the corresponding content of the polyphenols in the original samples.

$$\mathrm{Bioavailability}\;\left(\%\right)=\frac{{\mathrm{SPLPs}}_{A}}{{\mathrm{SPLPs}}_{T}}\times 100\%$$

where SPLPs_A_ indicates the SPLP compounds outside the dialysis membrane, and SPLPs_T_ represents the SPLP compounds in the supernatant after intestine digestion.

### 2.5. Bioactivity Evaluation

#### 2.5.1. Antioxidant Capacity

Digested SPLP antioxidants were comprehensively evaluated by the free radical scavenging capacity (DPPH^+^ and reactive oxygen species) and the reduction capacity (ferric-reducing antioxidant power capacity, FRAP). The results were expressed as μmol Trolox/g DW of SPLs.

The free radical scavenging activity was partly measured by the DPPH assay previously described by Rufino et al. [20] with minor modifications. Briefly, the samples were diluted to a certain concentration for working, and 2 mL DPPH (60 µg/mL) was mixed with 1 mL solution. The absorbance was measured after 30 min incubation at 517 nm, and calculated according to a Trolox (0~220 μmol) standard curve.

The FRAP assay was slightly modified from previous reports [20]. Briefly, FRAP reagents were prepared by mixing 20 mM FeCl_3_, 300 mM sodium acetate and 10 mM TPTZ solution in the dark (10:1:1, *v*/*v*/*v*). Then, 40 μL of sample or standard antioxidants (10–180 μmol) was added to a 96-well plate together with 280 μL of FRAP. The absorbance was further measured by a microplate reader (Spark, Tecan Laboratory Equipment Co., Shanghai, China) at 593 nm after incubation at 37 °C for 10 min.

The ORAC of the samples was determined according to Sun et al. with minor modifications [16]. Briefly, 25 µL of appropriately diluted samples was added to a microtiter plate and subsequently mixed with 150 µL of 1 µmol fluorescein sodium salt and 25 µL of 250 mM AAPH. For blank and standard wells, the samples were replaced by equivalent phosphate buffer or Trolox solution (10–180 µmol), respectively. All reaction wells measured the fluorescence intensity at 5 min intervals (Ex. 485 nm, Em. 538 nm) for 80 min after incubation, the ambient temperature should be maintained at 37 °C during both incubation and measurement. Results were calculated using the difference of area under the fluorescence burst curve between the blank or sample.

#### 2.5.2. Antihyperglycemic Activity

The α-amylase and α-glucosidase inhibitory activity of intestinal chyme was examined according to the previous report described by Flores et al. and Zeng et al., respectively, with minor modifications [21,22]. Briefly, the sample was diluted into the same concentration gradient, and acarbose (0.025–0.1 mg/mL) was used as a positive control. For the α-amylase inhibition assay, α-amylase was dissolved in PBS at pH 6.8 to a concentration of 0.1 U/mL. A volume of 250 μL of enzyme solution was mixed thoroughly with an equal volume of inhibitor and the total volume was made up to 1 mL with PBS, then incubated for 15 min at 37 °C. Subsequently, 250 μL of 1% (*w*/*v*) starch was added, and the reaction was carried out at 37 °C for another 15 min. Eventually, 500 μL DNS was added and boiled for 10 min. The absorbance of the mixture was measured at 540 nm (A_1_). The A_2_ and A_0_ groups used PBS as a substitute for enzyme solution and inhibitor, respectively. The A_3_ group used 500 μL PBS instead of enzyme solution and substrate.

For the α-glucosidase inhibition assay, the enzyme was dissolved in PBS at pH 6.8 to a concentration of 0.1 U/mL. A volume of 20 μL of enzyme solution was mixed thoroughly with 10 μL of inhibitor and the total volume was made up to 140 μL with PBS, then incubated for 15 min at 37 °C. Subsequently, 20 μL of 5 mmol *p*NPG was added, and the reaction was carried out at 37 °C for another 15 min. Eventually, the absorbance of the mixture was measured at 405 nm by a microplate reader (A_1_). The A_2_ and A_0_ groups used PBS as a substitute for enzyme solution and inhibitor, respectively. The A_3_ group used 140 μL PBS instead of enzyme solution and substrate. The calculation formula was as follows:

$$\mathrm{Inhibitory}\;\mathrm{activity}\;\left(\%\right)=\frac{A_{1}{-A}_{2}}{A_{0}-A_{3}}\times 100\%$$

### 2.6. Statistical Analysis

The obtained data were analyzed by using IBM SPSS version 25.0 (IBM, Armonk, NY, USA), and the significance analysis of means was performed by the Tukey test. Origin Pro 2021 (OriginLab, Northampton, MA, USA) and Adobe Illustrator version 28.0 (Adobe, San Jose, CA, USA) were used for further processing or plotting.

## 3. Results

### 3.1. Chemical Composition Analysis

**Figure 1:**
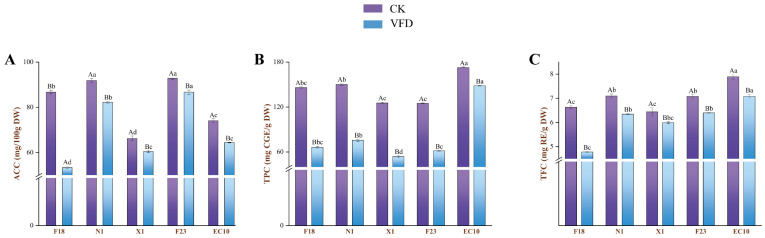
ACC (**A**), TPC (**B**) and TFC (**C**) of the samples. Values were the means ± standard deviation, n = 3. The different uppercase and lowercase letters indicate significant differences between varying indexes or drying methods and species, respectively, by the Tukey test (*p* < 0.05).

To understand the basic components of SPLs, the chemical composition of five varieties of SPLs was first analyzed in this study, and the results are shown in Figure S1. Briefly, the SPLs had a high protein content along with a fat content averaging 2.60 mg/g DW, which exhibited potential as a functional food ingredient. However, it was worth noting that the average moisture content in SPLs of more than 50% could pose challenges to their storage and processing. Thus, it was essential to prevent water leaching of polyphenol compounds by promptly undergoing post-harvest drying. To our knowledge, freeze-drying still seems to be one of the most effective techniques to retain bioactive compounds in leafy vegetables, even though drawbacks such as low efficiency and high energy consumption limit application [23,24,25,26]. Hence, this study further determined the content variations of ascorbic acid, total polyphenols and flavonoids that related to the biological activity of SPLs during the drying process (Figure 1). ACC, TFC, and TPC in SPLs decreased to varying degrees after the drying process, and the degradation rate of TFC was the most obvious decline compared to TPC or ascorbic acid, the TPC and TFC contents of EC10 were 172.86 mg CGE/g DW and 7.89 mg RE/g DW, respectively, while after drying, their retention rate was 14.08% and 10.27%, respectively, making it the most protective of the five varieties against phenolic compounds. For ACC, F23 showed a relatively higher content and retention rate after drying, which was 92.58 mg/100 g DW and 93.7%, respectively.

### 3.2. Phenolic Identification

**Figure 2:**
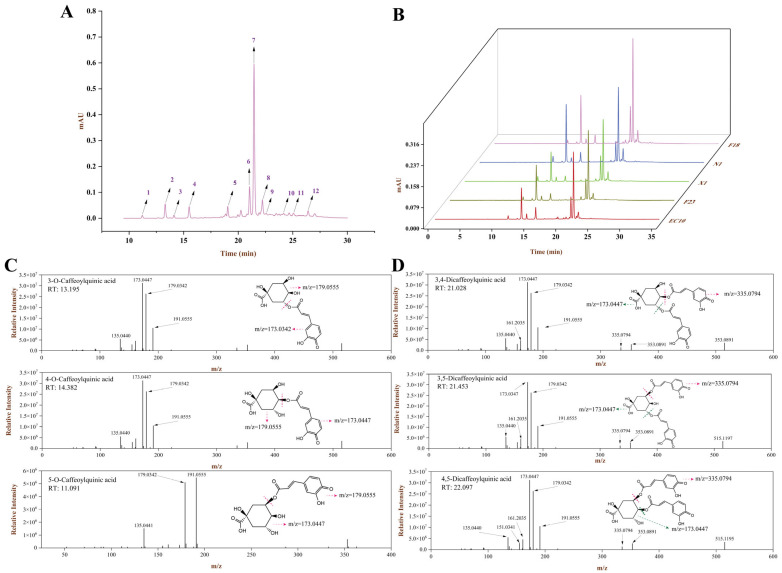
Phenolic profile and HRMS fragmentation of SPLPs. (**A**) Representative chromatograms of the identified SPLPs: (1) 5-CQA; (2) 3-CQA; (3) 4-CQA; (4) CA; (5) quercetin-3-O-hexoside; (6) 3,4-CQA; (7) 3,5-CQA; (8) quinine acid; (9) 4,5-CQA; (10) 3-caffeoyl-4-feruloylquinic acid; (11) quercetin; (12) 3,4,5-CQA. (**B**) The phenolic profile of five SPLs species. The MS^2^ fragmentation of 3-, 4- and 5-O-CQA (**C**) as well as 3,4-, 3,5- and 4,5-diCQAs (**D**).

To further investigate the difference between various SPLs of monomeric compounds and the changes in the simulated digestion process, UPLC-HRMS was used to identify phenolic compounds. In this study, twelve major components isolated in the chromatogram were further determined by HRMS, presented in Table S1 and Figure 2. The phenolic compositions of the five SPLs were basically the same, and the content of diCQAs was significantly higher than that of mono-CQAs. Among them, EC10 contained a higher amount of 3,4-, 3,5- and 4,5-diCQA, which were 1.88, 3.54, and 1.25 mg/g, respectively, confirmed by the results obtained in TPC analysis.

As described above, SPLs contain substantial amounts of caffeoylquinic acid isomers, while the 3-, 4- and 5-O-CQA had similar ion fragmentation in the negative ion mode, i.e., *m*/*z* 135, *m*/*z* 179, and *m*/*z* 191, which makes it difficult to simply analyze the ion fragments. The MS^2^ fragments of 4-O-CQAs were found to have higher relative intensities, and this outcome was similar to the description by Clifford et al. [27]. They claim to have successfully characterized mono-CQAs by retention time order and fragment intensity differences, but the reasons for such discrepancies were still unclear. For diCQAs, *m*/*z* 515 with strong relative intensity appeared in the full-scan mode of the primary MS, and those fragments were extracted for further MS^2^ scanning at the same fragmentation voltage; the results are shown in Figure 2D.

### 3.3. Bioaccessibility and Bioavailability Variations during Digestion In Vitro

#### 3.3.1. Bioaccessibility of TPC

**Figure 3:**
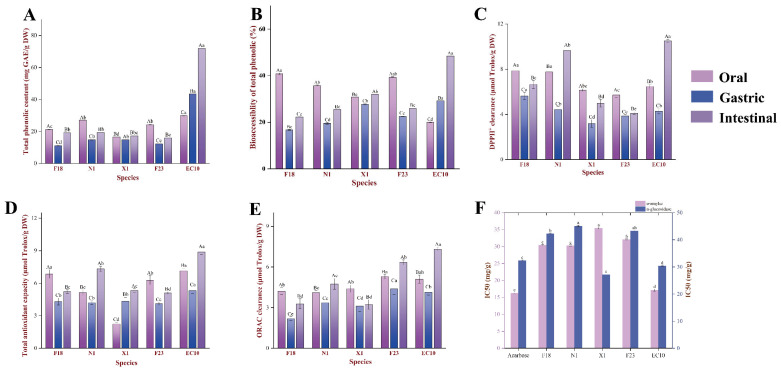
Effects of digestion on (**A**) TPC, (**B**) the bioaccessibility of TPC, (**C**) DPPH^+^ scavenging activity, (**D**) FRAP activity, (**E**) ORAC clearance capacity, and (**F**) α-amylase and glucosidase inhibitory activity. Different lowercase and uppercase letters represent significant differences (*p* < 0.05) in species and each digestive stage, respectively.

Food types and cooking methods could affect the profile and/or content of phenolic compounds, thus the concept of bioavailability was proposed, which refers to the proportion of active ingredient released from its substrate during the GI digestion and consequently exerting biological activity in the target organ [28,29]. As shown in Figure 1C and Figure 3A, the TPC after in vitro digestion was significantly lower than the original. Overall, the changes in TPC in digested extracts exhibited two different trends, (i) intestinal > oral > gastric; (ii) intestinal > gastric > oral. Typical of trend (i) was X1, which had 27.07, 14.77 and 19.35 mg GAE/g DW of TPC in the oral, gastric and intestinal phases, respectively, during oral-GI digestion, whereas the only EC10 that conformed to trend ii had a higher TPC in the intestinal phase compared to the oral and gastric phases by 141.21% and 65.81%, respectively. Together, these indicated that higher concentrations of phenolic compounds in EC10 would have the opportunity to enter circulation through the epithelial cells and achieve biological activity.

#### 3.3.2. Bioaccessibility and Bioavailability of Phenolic Profile after Digestion

As shown in Table 1 and Table S2, phenolic compounds were gradually released from SPLs as the digestion process progressed. Caffeic acid (CA) demonstrated relatively higher bioaccessibility at each phase, e.g., 12.17%, 69.01% and 29.45% in the oral, gastric and intestinal phases of EC10, respectively. Further, it was noteworthy that the bioaccessibility of CA in N1 was 274.16% during the oral stage, and 383.68% in the intestinal phase of EC10, suggesting the degradation of CQAs may have occurred during digestion. Such inference is also evidenced by the fact that the content of quinine acid was raised during the digestion process. Among the mono-CQAs, 4-O-CQA was initially undetectable in the oral and/or gastric phase, but presented in the intestinal stage. In contrast, the release behavior of diCQAs during digestion was rather different, with approximately equal amounts of diCQAs released at each stage, while the 3,4-, 3,5- and 4,5-diCQA released from EC10 during the intestinal stage were 9.32, 31.63 and 7.17 μg CGE/g DW, respectively, which led to the bioaccessibility of 6.55%, 0.27% and 13.18%, respectively.

After dialysis, each type of CQA that existed in intestinal juice could penetrate the dialysis membrane, albeit at relatively lower concentrations (Table 1 and Table S2). Among them, the dialysable CQAs content in EC10 was significantly higher than other species, and the concentration of 3-, 4- and 5-O-CQA were 112.93, 118.56 and 127.03 μg CGE/g DW, respectively, thus making mono-CQAs in EC10 relatively more bioavailable than F18. Nevertheless, we also noticed that the bioaccessibility of 4- and 5-O-CQA in N1, X1, and F23 was significantly higher than EC10 (*p* < 0.05), even though the content of these compounds in the dialysis medium was much lower, whereas the permeability of diCQAs and triCQAs was not satisfactory. For instance, the proportion of 3,4-, 3,5- and 4,5-diCQA in N1 that was able to penetrate the dialysis membrane was 40.78%, 34.07%, and 78.88%, respectively, although its superior mono-CQAs bioavailability was observed. And the bioavailability of the above components in EC10 was 62.98%, 89.47% and 67.36%, respectively, which was still significantly better than other species (*p* < 0.05). For 3,4,5-triCQA and CA, the bioavailability of their different species was essentially similar—they were dialysable in the range of 60.12–78.36%, except for 3,4,5-triCQA in F18 (47.25%).

### 3.4. Antioxidant Activity Analysis

Previous studies demonstrated that SPLPs usually exhibit powerful antioxidant properties and certain antiglycemic activity in mice [4,30]. Figure 3C illustrated a significant increase in DPPH^+^ scavenging activity throughout oral-GI digestion (*p* < 0.05). The antioxidant activity of the five SPLs generally decreased in the following order: intestinal > oral > gastric, which was similar to the changing pattern of TPC during digestion. Among the five SPL varieties chosen for this study, EC10 still had favorable antioxidant activity with DPPH, FRAP and ORAC was 10.51, 8.89 and 7.32 μmol Trolox/g DW in intestinal chyme, respectively. It was worth noting that the changes in antioxidant activity of EC10 and X1 did not follow the same trend as TPC dose, where the former showed 31.28% higher TPC content in the gastric than oral, but the antioxidant activity of gastric were declined to varying extents (DPPH, FRAP and ORAC were reduced by 51.17%, 39.40% and 23.48%, respectively), while the FRAP of the latter was significantly different with TPC.

### 3.5. Anti-Glycemic Activity Analysis

Considering that α-amylase and α-glucosidase usually play a role in the catabolism of carbohydrates during the intestinal digestion phase, analyzing the inhibitory effect of SPLPs on the above-mentioned enzyme activities contributes to the systematic study of the potential effects of SPLs on postprandial glucose. To simplify the system, other factors in the system such as expected polyphenols that may affect enzyme activity were not analyzed in this study, including resistant proteins or starch as mentioned previously. As shown in Figure 3F, although the enzyme inhibitory activities of SPLs were reduced after digestion, the α-amylase inhibitory activity of the entero-digestive fluid was stronger than that of α-glucosidase in all SPLs except X1. The α-amylase inhibitory activities of the five SPLs could be ranked as EC10 > F18 > N1 > F23 > X1, and a similar trend was observed in the inhibitory activities against α-glucosidase. In detail, EC10 had relatively stronger enzyme inhibitory activity, with IC50s of 19.36 mg/g and 25.21 mg/g for α-amylase and α-glucosidase, respectively, with more than 90% activity similar to the positive control.

## 4. Discussion

The evidence suggested that low moisture content contributes to the stabilization and extraction of phenolic compounds in the phyllosphere [31]. A further possible explanation was carbohydrates (mainly polysaccharides) and proteins could have covalent and/or non-covalent complexes with polyphenols, thus providing excellent stability to the products against adversity [32]. In this sense, EC10, with the lowest moisture content among the five SPLs in this study, was more suitable for the food processing industry due to the greater retention of TPC and TFC after drying.

**Figure 4:**
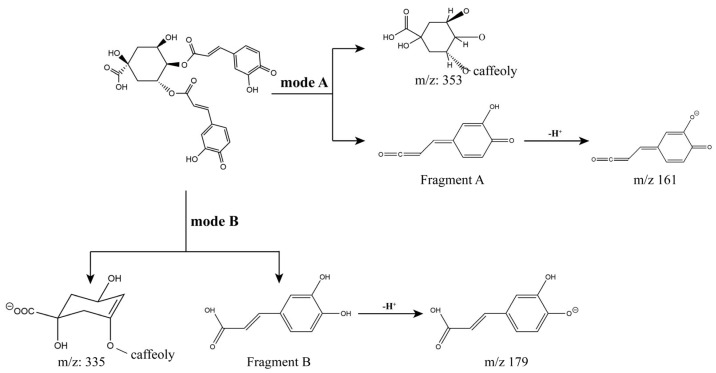
Presumed MS^2^ cleavage patterns of 3,4-diCQA.

In this study, compounds typically have several defined breakage patterns in MS^2^. Paull et al. reckoned the ester group in the molecule tends to dissociate and therefore breaks preferentially during the fragmentation process, which occurs in acyl-oxygen and/or alkyl-oxygen bond-breaking patterns [33]. Thus, we could infer that the ester group cleavage process also occurs in diCQAs. The superior bioactivity of diCQAs in SPLPs has now been well established, with activities such as antioxidant, antidiabetic, antihyperlipidemic activity and even anticancer properties [34,35,36,37], of which 3,5-diCQA exhibited the strongest potential activity [38]. As shown by UPLC-HRMS analysis, the abundance of 3,5-diCQAs (peak 7) in EC10 makes them become another promising source of high-quality polyphenols (Figure 2A,B), which might provide a viable solution for the intensive processing of SPLs. Further, these diCQAs appear to have both *m*/*z* 335 and 353 fragments during MS^2^ scanning, predicting that there may be more than one cleavage mode. Hence, we could summarize two possible paths of diCQAs cleave and presented in Figure 4. Taking 3,4-diCQA as an example, path-A initially dissociates the 4-position of caffeoyl to produce m/z 353 and a neutral fragment (A), and the latter could further deprotonate to generate *m*/*z* 161; path-B was the cleavage of the alkoxide bond, which caused the 4-position caffeoyl group ionized (*m*/*z* 335) and neutral fragment B formed, and the latter could result in the *m*/*z* 179. Combined with retention times in chromatographic, these MS^2^ fragmentation features on mono-CQAs and diCQAs enabled researchers to efficiently characterize caffeoylquinic acid isomers present in other phytomasses.

By analyzing the performance of SPLs in digestion, two divergent trends of the TPC were observed. Firstly, during the simulated digestion of F18, N1, X1 and F23, TPC was significantly reduced in the gastric phase compared to the oral phase. This could be explained by the combination of polyphenols with enzymes and plant protein through covalent or non-covalent interactions, as well as the reduced rate of trypsin-induced hydrolysis [39]. Polyphenol–macromolecule complexes obscure the UV absorption of the benzene ring, resulting in the inability to analyze the polyphenol concentration in the digested chyme by spectroscopic methods. Moreover, the lower pH of the gastric phase favors polyphenol solubilization and stability, which provides the foundation for polyphenol release during the intestinal phase of digestion [40,41]. With digestion proceeds and pH variation, phenolics were released due to the degradation of protein/carbohydrates–phenolic complexes and macromolecules, which explained the significant (*p* < 0.05) increase in TPC during the intestinal phase (Figure 3A,B). Previous studies suggested that dietary polyphenols were not fully released during GI digestion due to the inhibitory effects of polyphenols, resistant starch and proteins on digestive enzyme system activity [42]. Nevertheless, in response to the bioaccessibility of EC10 exhibited significant differences (*p* < 0.05), we presume that this might relate to the differences in their polysaccharide and protein composition. Precisely, since the EC10 matrix contains more proteins and carbohydrates than other species, this may make it contain a high amount of the polyphenol–protein/polysaccharide complexes, thus rendering it more resistant to enzymatic catabolism during the early stages of digestion. As oral-GI proceeded, the continued decomposition by amylases and glycosidases promoted the dissociation of the complexes, as evidenced by the continued increase in TPC content. In conclusion, statistics suggested that EC10 may have better polyphenol release characteristics, while further examination of the changes in the phenolic profile of SPLs during oral-GI is still required

Juániz et al. reported the degradation of diCQAs to mono-CQAs in both simulated and human intestinal medium [43], and this may explain the fact that the releasing quantity of the three diCQAs in digestion stages was still lower than the amount corresponding to the original samples. Further, the released concentration of CA and quinine acid (precursors of CQAs) far exceeded the original, indicating that further degradation in CQAs occurred. The vanishing of 4-O-CQA in the gastric digest of EC10, F23 and X1 reappearing in intestinal digestion may since polyphenol partly existed in a bound state and was released by trypsinization [44]. Jakobek et al. reckoned 4-O-CQA could be isomerized to 3-O-CQAs at higher pH (6–9) [45], whereas this compound became fully detectable in intestinal digestion after extraction with ethyl acetate in our study, which was similar to the result reported by Laurent et al. [46]. Thus, we believe this unique phenomenon may be due to the interaction between 4-O-CQA and the crosslinking of digestive enzymes with the caffeoyl group increases the stability of polyphenols while changing their spectral characteristics, which made 4-O-CQA barely undetectable in chromatographic analysis without extraction. Since diCQAs were more susceptible to isomerization or degradation at higher ambient temperatures and alkaline pH environments [47], which resulted in the contents of 3,4-, 3,5- and 4,5-diCQA detected in the intestinal fluid of EC10 were only 6.5%, 0.27% and 13.17% of the original, even the release of digestive enzymes already resulted in the dissolution of large amounts of polyphenols. Meanwhile, the content of CA increased by 363.36%, which can be considered as the dissociation of CQAs from proteins increased the concentration of polyphenols in the system, followed by degradation in alkaline pH [48]. In addition to the possible degradation reason of CQAs, the bioaccessibility was attributable to the varying water solubility of these compounds, thus made it difficult for the poorly water-soluble diCQAs to present abundantly in free form compared to mono-CQA within the digestive solution [49]. This also explains the lower bioavailability of diCQAs in the dialysis fluid. In addition, since diCQAs were better hydrogen donors compared to mono-CQAs due to more hydroxyl group content, they were more likely to cross-link with proteins in the digestive solution and not be able to through the dialysis bag. This results in differences between CQAs in the intestinal juice and dialysis fluid.

In summary, significant variances in the content and phenolic profile were found in the digestive juice of SPLs, which could be attributed to the digestive enzymes’ properties as well as other physiological environment indexes, including pH, ionic strength, and digestion time [50,51]. The changes in phenolic profile due to the release of water-soluble phenolics by amylase during oral digestion, but considering the brief duration of oral digestion, the effect of the oral phase on the in vitro digestion of phenolics could be disregarded for now [30]. During gastric digestion, the peptide chains were hydrolyzed by pepsin, contributing to the release of mono-CQAs and large amounts of diCQA, which were accompanied by the degradation of CQAs (CA bioaccessibility > 100%). During intestinal digestion, lipases, proteases and amylases from pancreatic as well as bile extracts were further hydrolyzing the cellular structure of SPLs, resulting in elevated content of free mono-CQAs. Our statistics suggested that SPLPs could be released by in vitro digestion thus generating biological activity in the GI environment. Furthermore, the release of CQAs was clearly influenced by the chemical environment and the complex reactions between in vitro digestive enzymes and SPLs matrix. This is typified by EC10, whereby significantly higher bioavailability is achieved compared to other varieties of SPLs. Considering that SPLs did not differ in polyphenol composition (only content differences, Figure 3B) or digestion treatment, we hypothesized that the complexes formed by proteins and/or polysaccharides with polyphenols in the EC10 were more resistant to low pH environments, while tended to undergo complete hydrolysis in high pH condition, enabling the free CQAs to achieve higher bioavailability. However, due to the lack of identification and characterization of proteins and polysaccharides in SPLs, this conjecture still needs to be substantiated by relevant studies.

The antioxidant mechanism of CQAs generally relates to pH because of the existence of the caffeoyl group. Specifically, polyphenols are easily deprotonated at ambient pH of neutral or below, which facilitates the antioxidant capacity of polyphenols, and this partially explains the higher antioxidant activity in the intestinal and oral stage digestive products compared to the gastric [52]. In addition, Ma et al. suggested that the increased release and isomerization of polyphenols may related to the increased antioxidant activity [53], which was consistent with the phenomenon obtained in our study. Phenolics were usually present in plants with the polysaccharides-bond mode, which limits the hydroxyl radical flexibility in the phenolic, thus reducing free radical scavenging or reducing ability [54]. Hydrolysis of the food matrix by amylase, protease and trypsin during digestion led to the loss of these conjugated structures, then increased the explanations of the structural, which leads to the rebound in antioxidant activity [55]. However, the bioactivities do not necessarily follow the same trend due to the different phenolic profiles caused by species differences, thus targeted analyses were still needed to clarify the product activity.

As for anti-glycemic activity, both α-amylase and α-glucosidase could degrade polysaccharides into simpler oligosaccharides or monosaccharides to make them available for intestinal absorption, then further increase glycemia levels [56]. Therefore, by inhibiting these two digestive enzymes could be a potential strategy for one of the diabetes therapies. Researchers similarly found that α-glucosidase inhibitory activity decreased significantly after digestion [57], Silva et al. reckoned that isomerization of phenolic acids driven by intestinal enzymes and pH reduces their α-amylase and glucosidase inhibitory activities [58]. Another possible explanation was the polyphenol-enzyme complexes formed during digestion, thus resulting in the lower enzyme inhibition activity contributed by the dissertation of free polyphenol content in samples [59]. In conclusion, our results indicated that the anti-glycemic activity of SPLs diminished with in vitro digestion, but was accompanied by partly increased antioxidant activity.

## 5. Conclusions

Distinctly different chemical compositions, polyphenol release properties, and biological activities were exhibited by five varieties of SPLs in this study. Among them, the superior ACC, TFC and TPC were presented in EC10, which exhibited its excellent nutritional value. Twelve polyphenol compounds in SPLs were identified by UPLC-HRMS, which were predominantly CQAs, and the MS^2^ spectra of diCQAs presented two distinct cleavage patterns. By further examining the changes in the phenolic profiles of SPLs during digestion, 3-, 4- and 5-O-CQA in the EC10 dialysis medium were 112.93, 118.56 and 127.03 μg CGE/g DW, respectively, which had better bioavailability and bioaccessibility than multisubstituted CQAs. Further, EC10 intestinal digestive juice had excellent DPPH, FRAP, and ORAC of 10.51, 8.89 and 7.32 μmol Trolox/g DW, respectively, but the digestion also reduced the inhibitory ability against the digestive enzymes. In summary, EC10 has the ability to be used as an ingredient for developing functional products, but the degradation mechanism, stability and in vivo bioactivity of diCQAs after digestion still need more in-depth research.

## Figures and Tables

**Table 1:** Bioaccessibility and bioavailability of the major polyphenol profile in SPLs during oral-GI digestion in vitro. nd, not detected; different lowercase letters indicate a significant difference (*p* < 0.05) between species at the same conditions.

		CQAs							Phenolic	
		3-CQA	4-CQA	5-CQA	3,4-CQA	3,5-CQA	4,5-CQA	3,4,5-CQA	CA	Quinine Acid
Oral (Bioaccessibility %)	F18	0.58 ^b^	nd	nd	0.72 ^c^	0.34 ^c^	1.34 ^b^	nd	15.41 ^b^	100.00 ^a^
	N1	0.41 ^c^	nd	1.90 ^c^	0.97 ^b^	0.34 ^c^	0.74 ^c^	0.62^c^	274.16 ^a^	nd
	X1	0.51 ^b^	nd	2.52 ^b^	nd	2.70 ^a^	2.54 ^b^	nd	4.81 ^d^	nd
	F23	0.86 ^a^	3.54 ^a^	3.99 ^a^	1.22 ^a^	0.39 ^b^	4.08 ^a^	7.45 ^a^	3.25 ^e^	nd
	EC10	0.55 ^c^	nd	2.48 ^bc^	nd	0.32 ^c^	2.73 ^b^	4.38 ^b^	12.17 ^c^	nd
Gastric (Bioaccessibility %)	F18	0.27 ^d^	nd	3.75 ^b^	nd	0.14 ^d^	1.48 ^d^	3.89 ^c^	49.01 ^b^	100.00 ^a^
	N1	0.81 ^b^	3.45 ^a^	3.36 ^b^	1.09 ^a^	0.29 ^c^	2.49 ^b^	6.54 ^b^	1.25 ^e^	nd
	X1	nd	nd	nd	nd	0.45 ^a^	3.99 ^a^	9.01 ^a^	23.19 ^c^	nd
	F23	nd	nd	nd	0.99 ^a^	0.43 ^a^	1.81 ^c^	nd	16.15 ^d^	nd
	EC10	0.97 ^a^	nd	6.57 ^a^	nd	0.34 ^b^	3.84 ^a^	6.83 ^b^	69.01 ^a^	100.00 ^a^
Intestinal (Bioaccessibility %)	F18	0.97 ^c^	nd	3.70 ^b^	2.20 ^b^	0.25 ^c^	3.22 ^c^	6.88 ^a^	4.16 ^c^	nd
	N1	0.67 ^d^	1.32 ^c^	1.81 ^d^	1.30 ^c^	0.47 ^a^	3.79 ^b^	6.88 ^a^	12.90 ^b^	nd
	X1	0.52 ^e^	3.40 ^b^	2.96 ^c^	1.20 ^d^	0.36 ^b^	3.86 ^b^	nd	nd	nd
	F23	1.34 ^b^	3.54 ^b^	3.26 ^bc^	1.08 ^d^	0.47 ^a^	1.76 ^d^	nd	nd	nd
	EC10	9.75 ^a^	57.39 ^a^	79.37 ^a^	6.55 ^a^	0.27 ^c^	13.18 ^a^	5.12 ^b^	383.68 ^a^	nd
Bioavailability (%)	F18	45.34 ^b^	nd	61.08 ^b^	39.54 ^d^	nd	36.62 ^c^	47.25 ^b^	78.36 ^a^	nd
	N1	57.67 ^a^	62.16 ^b^	80.74 ^a^	40.78 ^d^	34.07 ^c^	78.88 ^b^	60.12 ^ab^	73.20 ^b^	nd
	X1	55.85 ^ab^	66.23 ^b^	83.11 ^a^	68.31 ^b^	41.68 ^b^	69.43 ^c^	nd	nd	nd
	F23	46.47 ^b^	75.32 ^a^	86.24 ^a^	60.90 ^c^	40.39 ^b^	73.31 ^bc^	nd	nd	nd
	EC10	60.01 ^a^	68.86 ^b^	62.12 ^b^	79.13 ^a^	62.98 ^a^	89.47 ^a^	67.36 ^a^	76.35 ^a^	nd

## Data Availability

The data presented in this study are available on request from the corresponding author.

## Supplementary Materials

The following supporting information can be downloaded at: https://www.mdpi.com/article/10.3390/antiox13050520/s1, Figure S1: The chemical composition of SPL; Table S1: Compounds identification, compound formula, retention times, measured *m*/*z* of molecular and mass fragments (MS^2^) in SPL; Table S2: Content changes in the phenolic profile in SPLs after each digestion stage. References [60,61,62,63] are cited in the supplementary materials.
