# Supplementary material for: In Vitro Gastrointestinal Digestion of Various Sweet Potato Leaves: Polyphenol Profiles, Bioaccessibility and Bioavailability Elucidation
Source: Antioxidants (Basel). 2024 Apr 26;13(5):520. doi: 10.3390/antiox13050520 (PMC11117659; doi:10.3390/antiox13050520)
Supplement: Supplementary file 1 [file antioxidants-13-00520-s001.zip › antioxidants-2925689-supplementary.pdf]

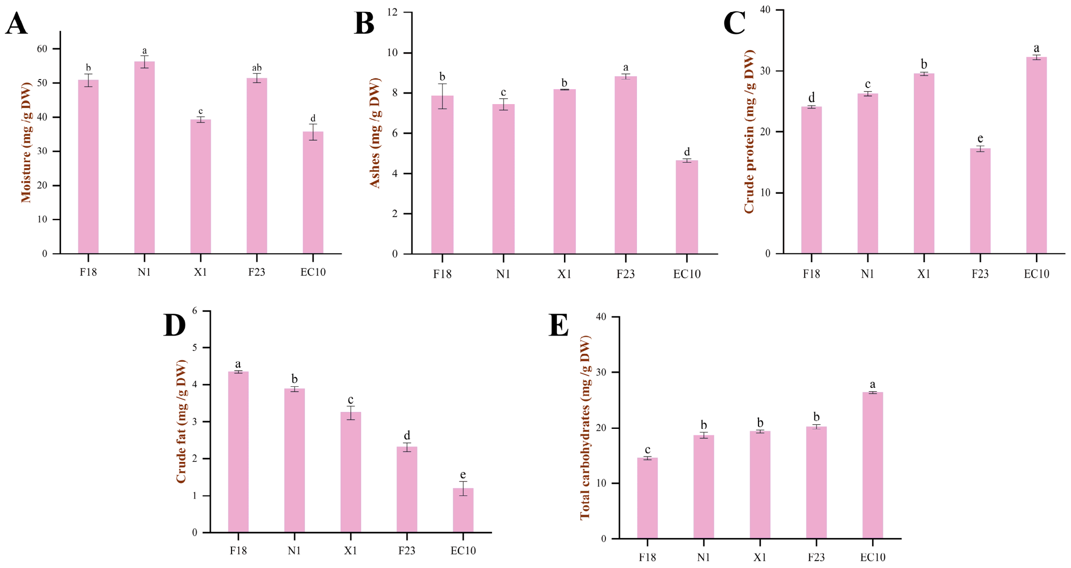

Figure S1 The chemical composition of SPLs, (**A**) Moisture; (**B**) Ashes; (**C**) Crude protein; (D) Crude fat; (E) Total carbohydrates. Values were mean ± standard deviation, n=3. The lowercase letters indicate significant differences between species, respectively, by the Tukey test ($p < 0.05$)

Table S1. Compounds identification, compound formula, retention times, measured m/z of molecular and mass fragments (MS^2) in SPL.

NO.	Compound	Rt [min]	Compound formula	Measured *m/z*	MS/MS *m/z*	Reference
1	5-O-Caffeoylquinic acid (5-CQA)	11.090	$C_{16}H_{18}O_9$	353.0886	191.0555, 179.0555, 173.0342, 161.0105, 135.0441	[1]
2	3-O-Caffeoylquinic acid (3-CQA)	13.195	$C_{16}H_{18}O_9$	353.0886	191.0555, 179.0555, 173.0447, 135.0441	[1]
3	4-O-Caffeoylquinic acid (4-CQA)	14.328	$C_{16}H_{18}O_9$	353.0886	191.0555, 179.0342, 173.0447, 135.0441	[1,2]
4	Caffeic acid (CA)	15.309	$C_9H_8O_4$	179.0339	135.0480, 134. 0152	[1,2]
5	Quercetin-3-O-hexoside	19.024	$C_{24}H_{22}O_{15}$	463.0458	300.0328, 301.0228, 271.0468, 255.0513, 179.0490	[3]
6	3,4-Dicaffeoylquinic acid (3,4-CQA)	21.028	$C_{25}H_{24}O_{12}$	515.1197	353.0891, 335.0794 ,191.0555, 179.0343, 135.0440	[1,3]
7	3,5-Dicaffeoylquinic acid (3,5-CQA)	21.453	$C_{25}H_{24}O_{12}$	515.1197	353.0891, 335.0794,191.0555, 179.0343, 135.0440	[1]
8	Quinine acid	21.788	$C_{11}H_9NO_3$	191.0561	191.0243, 85.0273, 126.8311, 93.3069, 87.1055	[1]
9	4,5-Dicaffeoylquinic acid (4,5-CQA)	22.097	$C_{25}H_{24}O_{12}$	515.1197	353.0749, 335.0794,191.0555, 173.0343 135.0440	[1]
10	3-Caffeoyl-4-feruloylquinic acid	24.086	$C_{26}H_{26}O_{12}$	529.1351	367.1043, 353.0910, 193.0535, 191.0573, 179.0412,	[1]
11	Quercetin	25.019	$C_{15}H_{10}O_7$	301.0354	151.0214, 121.0983, 107.0258, 179.0583	[1]
12	3,4,5-Tricaffeoylquinic acid (3,4,5-CQA)	26.483	$C_{34}H_{30}O_{15}$	677.1521	515.0723, 353.0328	[1,4]

Abbreviations: Rt (retention time)

Table S2. Contents changes of the phenolic profile in SPLs after each digestion stage, different lowercase letter indicates significant difference between species by the Turkey test ($p < 0.05$).

		**CQAs**							**Phenolic**	
		**3-CQA**	**4-CQA**	**5-CQA**	**3,4-CQA**	**3,5-CQA**	**4,5-CQA**	**3,4,5-CQA**	**CA**	**Quinine acid**
Undigested mg CGE /g DW	F18	1.66±0.04^{ab}	0.27±0.01^{a}	0.25±0.02^{b}	1.39±0.03^{b}	2.78±0.03^{b}	0.29±0.03^{b}	0.14±0.03^{a}	0.44±0.01^{b}	nd
	N1	1.46±0.03^{b}	0.28±0.03^{a}	0.33±0.04^{a}	0.83±0.03^{d}	2.71±0.01^{b}	0.26±0.02^{b}	0.14±0.01^{a}	0.14±0.05^{d}	nd
	X1	1.25±0.05^{c}	0.27±0.02^{a}	0.25±0.03^{b}	0.86±0.01^{cd}	2.54±0.03^{c}	0.25±0.01^{b}	0.14±0.02 ^{a}	0.39±0.01^{c}	nd
	F23	1.06±0.01^{d}	0.26±0.01^{a}	0.29±0.01^{a}	0.99±0.03^{c}	2.28±0.01^{d}	0.53±0.02^{a}	0.09±0.02^{b}	0.33±0.04^{c}	nd
	EC10	1.93±0.02^{a}	0.30±0.01^{a}	0.25±0.00^{b}	1.88±0.01^{a}	3.45±0.04^{a}	0.24±0.01^{b}	0.14±0.03^{a}	0.55±0.02^{a}	nd
Oral µg CGE /g DW	F18	9.63±0.04^{b}	nd	nd	10.01±0.03^{b}	9.45±0.01^{c}	3.89±0.02^{c}	nd	67.804±0.01^{b}	10.13±0.02^{a}
	N1	5.99±0.02^{c}	nd	6.27±0.03^{b}	8.05±0.01^{c}	9.21±0.03^{c}	1.92±0.04^{d}	0.87±0.02^{b}	383.82±0.03^{a}	nd
	X1	6.38±0.01^{c}	nd	6.30±0.02^{b}	nd	68.58±0.04^{a}	6.35±0.02^{b}	nd	18.76±0.02^{d}	nd
	F23	9.12±0.02^{b}	9.20±0.01^{a}	11.57±0.05^{a}	12.08±0.03^{a}	8.89±0.03^{c}	21.62±0.02^{a}	6.71±0.05^{a}	10.73±0.04^{e}	nd
	EC10	10.62±0.03^{a}	nd	6.20±0.04^{b}	nd	11.04±0.02^{b}	6.55±0.01^{b}	6.13±0.01^{a}	66.94±0.03^{c}	nd

Gastric µg CGE /g DW	F18	4.48±0.02^{c}	nd	9.38±0.03^{c}	nd	3.89±0.04^{d}	4.29±0.01^{c}	5.45±0.02^{c}	215.64±0.02^{b}	5.45±0.03^{b}
	N1	11.83±0.01^{b}	9.66±0.03^{a}	11.09±0.04^{b}	9.05±0.01^{a}	7.86±0.02^{c}	6.47±0.03^{b}	9.16±0.01^{b}	1.75±0.04^{e}	nd
	X1	nd	nd	nd	nd	11.43±0.03^{a}	9.98±0.04^{a}	35.14±0.02^{a}	90.44±0.01^{c}	nd
	F23	nd	nd	nd	9.80±0.01^{a}	9.80±0.05^{b}	9.59±0.02^{a}	nd	53.30±0.03^{d}	nd
	EC10	18.72±0.02^{a}	nd	16.43±0.01^{a}	nd	11.73±0.03^{a}	9.22±0.04^{a}	9.56±0.05^{b}	379.56±0.02^{a}	14.11±0.04^{a}
Intestinal µg CGE /g DW	F18	16.10±0.03^{b}	nd	9.25±0.02^{b}	30.58±0.02^{b}	6.95±0.04^{d}	9.34±0.01^{b}	9.63±0.03^{a}	18.30±0.05^{b}	nd
	N1	9.78±0.02^{d}	3.70±0.05^{c}	5.97±0.03^{d}	10.79±0.02^{c}	12.74±0.01^{a}	9.85±0.03^{b}	9.63±004^{a}	18.06±0.01^{b}	nd
	X1	6.50±0.02^{e}	9.18±0.02^{b}	7.40±0.01^{c}	10.32±0.04^{c}	9.14±0.03^{c}	9.65±0.01^{b}	nd	nd	nd
	F23	14.20±0.05^{c}	9.20±0.02^{b}	9.45±0.03^{b}	10.69±0.02^{c}	10.72±0.03^{b}	9.33±0.04^{b}	nd	nd	nd
	EC10	188.18±0.04^{a}	172.17±0.01^{a}	198.43±0.03^{a}	123.14±0.05^{a}	9.32±0.01^{c}	31.63±0.02^{a}	7.17±0.01^{b}	2110.24±0.03^{a}	nd
post-dialysis µg CGE /g DW	F18	7.30±0.01^{b}	nd	5.65±0.02^{d}	12.09±0.04^{b}	nd	3.42±0.03^{c}	4.55±0.03^{b}	14.34±0.02^{b}	nd
	N1	5.64±0.02^{d}	0.50±0.03^{d}	4.82±0.04^{d}	4.40±0.04^{d}	4.34±0.05^{b}	7.77±0.02^{b}	5.79±0.05^{a}	13.22±0.01^{c}	nd
	X1	3.63±0.01^{c}	6.08±0.04^{c}	6.15±0.03^{c}	7.05±0.01^{c}	3.81±0.05^{b}	6.70±0.03^{b}	3.63±0.02^{c}	6.08±0.04^{d}	nd
	F23	7.62±0.03^{b}	6.93±0.04^{b}	8.15±0.01^{b}	6.51±0.02^{c}	4.33±0.03^{b}	6.84±0.02^{b}	nd	nd	nd
	EC10	112.93±0.02^{a}	118.56±0.05^{a}	127.03±0.04^{a}	97.44±0.03^{a}	5.87±0.01^{a}	28.30±0.02^{a}	4.83±0.05^{b}	1611.98±0.03^{a}	nd

Abbreviations: nd (not detected)

References:

1. Fu, Z.F.; Tu, Z.C.; Zhang, L.; Wang, H.; Wen, Q.H.; Huang, T. Antioxidant activities and polyphenols of sweet potato (Ipomoea batatas L.) leaves extracted with solvents of various polarities. *Food Biosci* 2016, 15, 11-18. doi: 10.1016/j.fbio.2016.04.004
2. Lu, X.F.; Zhou, Y; Ren, Y.P.; Zhang J. Improved sample treatment for the determination of flavonoids and polyphenols in sweet potato leaves by ultra performance convergence chromatography-tandem mass spectrometry. *J. Pharm. Biomed. Anal* 2019, 169, 245-253. doi:10.1016/j.jpba.2019.03.003
3. Sun, R.; Kan, J.; Cai, H.; Hong, J.; Jin, C.; Zhang M. In vitro and in vivo ameliorative effects of polyphenols from purple potato leaves on renal injury and associated inflammation induced by hyperuricemia. *J Food Biochem* 2022, 46(2), e14049. doi: 10.1111/jfbc.14049
4. Zhang, L.; Tu Z.C.; Wang, H.; Fu, Z.F.; Wen, Q.H.; Chang, H.X.; Huang, X.Q. Comparison of different methods for extracting polyphenols from Ipomoea batatas leaves, and identification of antioxidant constituents by HPLC-QTOF-MS2. *Food Res. Int* 2015, 70, 101-109. doi: 10.1016/j.foodres.2015.01.012
